# Latham on Diseases of the Heart

**Published:** 1847-02

**Authors:** 


### PART III.—BIBLIOGRAPHICAL NOTICES.

### ARTICLE X.

LATHAM *on Diseases of the Heart.* Forming the Oct. No. of Bell's Select Medical Library.

The learned lecturer it is to be hoped, from the size of his volume, has succeeded in throwing some additional light upon this obscure and too frequently complicated class of diseases. He devotes these lectures to the use of mercurials in inflammations generally, and diseases of the heart in particular. A subject susceptible of much useful investigation. Of the *manner* in which he has handled it, and the disorders of the organ, the subject of the volume, we are not prepared to speak, having given it but a cursory examination. We shall take occasion to refer to it again. G. N. F.

